# Linking oral health with other health domains among edentulous patients

**DOI:** 10.6026/973206300211391

**Published:** 2025-06-30

**Authors:** Mayur Mishra, Mandeepsinh Gohil, Gautam Rai, Jemin Borda, Sahil Khokhar, Rohan Shambhuwani

**Affiliations:** 1 Department of Public Health Dentistry, KM Shah Dental College and Hospital, Vadodara, Sumandeep Vidyapeeth Deemed to be University, Piparia, Vadodara, Gujarat, India; 2 Department of Public Health Dentistry, College of Dental Science, Amargadh, Gujarat, India; 3 Department of Oral Medicine and Radiology, College of Dental Science and Research Centre, Bopal, Ahmedabad, Gujarat, India; 4 Director, Diamond hospital, Bhavnagar, Gujarat, India; 5 Department of Prosthodontic crown and bridge, College of Dental Science, Amargadh, Gujarat, India

**Keywords:** Edentulous, nutrition status, psychological status oral health impact profile-edentulous (OHIP-EDENT), mini nutritional assessment (nestle 2009), beck's depression inventory (BDI)

## Abstract

Nutritional, emotional dimensions and oral health related quality of life among edentulous patient using Oral health impact profile-edentulous (OHIP-EDENT), Mini nutritional Assessment (Nestle 2009) and Beck's Depression Inventory (BDI) is of interest. The response showed there are 44.8% of participants at risk of malnutrition. And 23.6% diagnosed with malnutrition. Beck's depression inventory found out that around 23.6% to 12.4% had symptoms of mild to moderate depression. Gender wise Comparison of Variables done to find out association between them, it showed only OHIEDENT score were found to be significant. Based on the findings of this study, it was concluded that the self-reported quality of life can be influenced by the tooth loss.

## Background:

Oral health is a key indicator of overall health, well-being and quality of life. It encompasses a range of diseases and conditions that include dental caries, periodontal (gum) disease, tooth loss, oral cancer, oro-dental trauma, noma and birth defects such as cleft l ip and palate. Most oral diseases and conditions share modifiable risk factors with the leading non-communicable diseases (cardiovascular diseases, cancer, chronic respiratory diseases and diabetes). There is a proven relationship between oral and general health [1]. One of the common oral problems associated with advancing age is tooth loss [2]. Tooth loss implies in loss of several orofacial structures, such as bone tissues, nerves, receptors and muscles and consequently, most orofacial functions are diminished. Studies have indicated that the decrease of number of teeth and for complete denture wearers, the chew's ability is significantly less efficient. This may have consequences over general health and QoL of those patients [3]. Oral health is an integral part of general health and edentulism does affect the overall well-being of an individual [2]. Edentulism is generally associated with ageing, although one can keep own teeth throughout all life. The rate of edentulousness differs between cultures and countries with a trend to decline in rich countries due to investments and efforts in education on oral hygiene maintenance and other preventive measures. However, the prevalence of edentulousness is still large, mostly in less developed countries [4]. Tooth loss can thus cause discomfort, pain and functional limitations which could lead to disability and finally, handicap; and may predispose to a feeling of depression. Loss of teeth can substantially affect oral and general health, enjoyment with food and overall nutrition, thereby affecting the quality of life as well as oral health-related quality of life (OHRQOL) [5]. Extensive tooth loss reduces chewing performance and affects food choice, leading to malnourishment. Conventional complete dentures are the most commonly used rehabilitation procedure for edentulous patients; however, dentures affect chewing ability as it requires more strokes and time to chew food. Decreased chewing performance, impact on taste and food selection with difficulty in swallowing as it becomes poorly coordinated, results in choking, low diet quality and poor overall general health. Malnutrition in the elderly because of poor food choices and intake of key nutrients, causing various nutritional problems has an evident impact on their general health and quality of life. The oral cavity is closely related to nutrition because it is the entrance to the digestive tract. There are relatively very few studies on oral health and nutritional status [6]. Dental status, among other factors, has an impact on the nutritional status of the elderly. Regarding the oral sphere, multiple factors, other than dental status, have an impact on nutrition: muscular force decreases as a function of age, leading to longer mastication and a decrease in the quantity of saliva has been observed with age, essentially for non-stimulated saliva, together with a modification of saliva composition. Therefore, the capacity to form a bolus to permit swallowing (plastic, cohesive and slippery) is jeopardised [7]. The body schema (the psychological image of physical self) is heavily invested with emotional meaning and significant changes in body image can result in varying degrees of emotional instability. The loss of teeth which causes many adverse anatomic, aesthetic and biomechanical sequelae can also prove to be a terrible psychological shock to patients [8]. India has around 100 million elderly at present and the number is expected to increase to 323 million, constituting 20% of the total population, by 2050 [6]. Prevalence of edentulousness in India is 19%-32% [9]. In order to fully understand "the burden of illness" among the edentulous patient group, one must, therefore, cultivate an understanding of the physical, as well as the psychosocial dimensions of tooth loss. Thus this study carried out to find out Nutritional, emotional dimensions and oral health related quality of life of edentulous patient. Therefore, it is of interest to evaluate the relationship between oral health related quality of life, mental health and nutritional status of edentulous patient.

## Methods:

A cross-sectional study was conducted among 250 edentulous patients at COLLEGE OF DENTAL SCIENCE & HOSPITAL, AMARGADH after obtaining ethical clearance from ethical committee of college. The study was conducted among edentulous persons with or without removable dentures above or 50 years of age who visited the dental college between October 2022 and December 2022; if with dentures, dentures not older than 2 years, without any debilitating illness. All the completely edentulous patients reporting to the dental college will be recruited for the study. The sample size was calculated based on the prevalence of edentulous patients using the following formula:

- Zα/2 P (1-P)/d2

(Zα/2- normal deviate, P: 19% prevalence (in India), D: 5% margin of error)

This gives sample size 237, which was decided to examine 250 participants

## All the participants were selected based on the following criteria:

## Inclusion criteria:

[1] Edentulous patient who are willing to give consent.

[2] Age Group: 50 or above 50 Years.

[3] Male & Female both are included.

## Exclusion criteria:

[1] Patient with debilitating disease and oral lesions.

[2] Patient with history of psychological disorder.

[3] Patient who were operated for cleft, oral cancer or other prosthetic surgery.

A nonprobability convenience sampling method was used and a predesigned questionnaire was used to collect the data.

All the participants after signing an informed consent, was asked to fill self-administered questionnaire, which included 4 parts:

[1] Demographic characteristics (age, sex, marital status, spouse-alive or dead, education, income, residence, medical problems and habits) and consent form.

[2] Oral health impact profile-edentulous (OHIP-EDENT)

[3] Mini nutritional Assessment (Nestle 2009)

[4] Beck's Depression Inventory (BDI)

## For the assessment of oral health-related quality of life (OHRQoL):

The patients' quality of life was assessed through the OHIP-EDENT-19 questionnaire, which allows the evaluation of the perception of oral health. All questionnaires were applied by a single examiner. The score was calculated by assigning points to the answers (0 = never, 1 = sometimes, 2 = almost always). OHIP-EDENT-19 response data were analysed in four domains:

[1] Chewing complaints (questions 1, 5, 10 and 11)

[2] Discomfort and psychological problems (questions 8, 9, 12, 13 and 14)

[3] Social incapacity (questions 15-19)

Oral discomfort and pain (questions 2-4, 6 and 7)

Lower scores indicate higher satisfaction of individuals' oral health and better quality of life.

OHIP-EDENT is a modification of OHIP for edentulous patients and it is a 19-item questionnaire with seven subscales: functional limitation, physical pain, psychological discomfort, physical disability, psychological disability, social disability and handicap [10].

## For the assessment of nutritional condition:

For the assessment of nutritional status, only the screening of the "Mini Nutritional Assessment" (MNA®) was used. The full assessment consists of different measurements (weight, height and weight loss in previous three months) combined with brief questions related to lifestyle, medication, mobility, diet and subjective assessment. Based on weight and height, the Body Mass Index (BMI) was calculated. The results were documented in a point score. The maximum for the applied screening is 14 points. A score ≥ 12 indicates an adequate nutritional status, a score between 8 and 11 shows a risk of malnutrition and a score ≤ 7 denotes malnutrition [11].

## For the assessment of mental health:

Beck's Depressive Inventory (BDI) - A 21-item questionnaire and each item has a response from 0 to 3. The total scores for the 21 items were summated and scores within 1-10 are considered normal and as the scores increase, the severity of depression increases [12]. The questionnaire had close ended responses. Most of the responses were mostly recorded on likert scale and glickmann scale. The instruments/questionnaire was validated and the examiners were trained and calibrated in the department. A pilot study on 20 patients following the inclusion and exclusion criteria was conducted. For test-retest reliability, the patients were given the questionnaire at two different occasions to check the reproducibility of the questionnaire. Internal consistency (Cronbach's alpha) for questionnaire was excellent. For the study, the denture /oral status was evaluated by a trained clinician. Data will be collected using short interviews which will be guiding the patients to fill the questionnaire by interviewer. Clinician also helped participant to comprehend the questions.

## Statistical analysis:

The data was summarised and analysed using Ms excel and SPSS 20. The mean scores of BDI, OHIP-EDENT and MNA scales was compared with demographic and edentulous variables using Categorical analysis using the Chi-square test has been used to assess the relationship between the variables. The odds ratio (OR) was calculated using multiple logistic regression (entry method) after adjusting for potential confounders. Pearson correlation test were used to finding association. The level of statistical significance was set at 5%.

## Results:

The study was conducted among 250 edentulous patients of 50-70 years old. There were 80 participants who were denture wearer since last two years the study included both the gender, male: female = 144:106. Other demographic details shows that there 28 (11.2%) had lost their spouse. There were only 17 % of participants who had education above metrication. 83% of the population belonged to rural area. Oral Health Impact Profile for edentulous patients OHIP-EEDENT score are presented in Table 1 (see PDF). Responses of mini nutritional assessment (nestle 2009) presented in Table 2 (see PDF), with their interpretation in Table 3 (see PDF). The response showed there are 44.8% of participants at risk of malnutrition. And 23.6% diagnosed with malnutrition (Table 3 - see PDF). Table 4 (see PDF) shows Interpretation of beck's depression inventory. The inventory found out that around 23.6% to 12.4% had symptoms of mild to moderate depression. Gender wise Comparison of Variables done to find out association between them, it showed only OHIEDENT score were found to be significant (Table 5 - see PDF). The Pearson correlation coefficient is used to measure the strength of a linear association between two variables, the value of R between OHIEDENT and MNA score is 0.4278. The value of R between OHIEDENT and BDI score is 0.3156. Although technically a positive correlation, the relationship between variables is weak (the nearer the value is to zero, the weaker the relationship) (Table 6 - see PDF, 7 - see PDF and Figure 1 - see PDF, 2 - see PDF).

## Discussion:

Edentulism is an oral condition that corresponds to a public health problem, because the dental loss is responsible for a series of alterations in the stomatognathic system. The edentulism has significant impact on the subject's quality of life, since tooth loss causes reduction of masticatory ability, aesthetic problems, phonetic alteration, as well as nutritional and psychological deficits [13]. The present cross sectional study was conducted on 250 edentulous patients in order to fully understand "the burden of illness" among the edentulous patient group, to cultivate an understanding of the physical, as well as the psychosocial dimensions of tooth loss. Thus this study carried out to find out Nutritional, emotional dimensions and oral health related quality of life of edentulous patient. To evaluate the relationship between oral healths's related quality of life, mental health and nutritional status of edentulous patient. In present study it was noted that male participants were having higher OHIEDENT score than female and it showed statistical significant. Most of the patients had suffered from difficulties in chewing (137 male +95 female). Other than that the patients profile shows that they were worried about their chewing ability (126 male +64 female). Some time they were upset and been embarrassed about the condition (138 male +74 female). This led them to certainly less enjoying than others (87 male +40 female). These symptoms were less common in patients with the dentures. It has been stated in literature that the nutritional condition deteriorates in edentulous patients. The chewing ability is associated with food choices. Impaired chewing ability implies poor food choices and it affects an individual's overall nutrition. It can play a key role in the onset and duration of depression [14]. The present study has tried to find out using six items scale Nutritional Status of individual (Mini Nutritional Assessment). MNA scale evaluated that around 31.6% edentulous had normal nutritional status. Though, it has shown that 44.8% participants were at the risk of malnutrition. Their diet and interest in food were altered after their loss of tooth. 23.6% edentulous patients were mal-nutritive or near to it. The same finding was analysed study done by Banerjee R on edentulous patients in Maharastra. A similar study done by Cousson *et al.* using MNA scale in French found to be in accordance with the present study [7]. The data from the present study were independent with their time span of edentulousness and socioeconomic status. Chew ability may also effluence dietary preferences and this may contribute for the patient's nutritional status. However, this is matter of discussion since masticatory ability and efficiency are not only factors affecting nutrition [3]. There are chances of confounding in these data as age, metabolism, systemic condition, socioeconomic status and other psychological also affect the diet and nutrition status of individual. Moreover, aging is a biologic process which leads to substantial personal difference in oral ability [15]. Though when these data were compared to OHIP score; it was found that there is chance that higher OHIP score are significantly associated with higher MNA score.

Odds ratios that is 1.64, greater than 1 indicate that the event is more likely to occur as the predictor increases (Table 6 - see PDF). Results of the Pearson correlation indicated that there is a significant medium positive relationship between OHIPEDENT on (y axis) and MNA (on x axis) (Figure 1 - see PDF). Impaired chewing ability, poor OHRQoL and its' impact on depression. Depression has been reported to predispose to oral diseases due to biological disturbances and a lack of motivation. Likewise, oral diseases have also been found to impair self-esteem, OHRQoL and thereby, affecting the psychological well-being of individuals [14]. To fully estimate the burden of illness due to edentulism and establish valid treatment outcomes measures in this regard, it is equally important to study its psychosocial repercussions. The psychological status of the edentulous Participant was examined in relation to their chewing abilities using evaluation depression status of individual. (Beck's Depressive Inventory) BDI is a 21-item questionnaire. It was found that 45.2% has no sign of depression. Where else the rest have shown mild to moderated sign of depression. These results were in accordance with the previous study done by Shah *et al.* in Gujrat [8]. It is stated has loss of tooth, appetite and inability to masticate are the reason for the depressive states. Those with difficulties accepts tooth loss had a greater effect on self-esteem and social life, had more reservation about discussing tooth loss and was more likely to experience depression [8]. But here also confounding factors role cannot be neglected. When these data were calculated for odds ration it showed participant with higher OHIP EDENT score were 2.92 times more likely to get higher BDI scores than the rest (Table 7 - see PDF). Results of the Pearson correlation indicated that there is a significant medium positive relationship between OHIPEDENT on (y axis) and BDI (on x axis). The value of R between OHIEDENT and MNA score is 0.4278. The value of R between OHIEDENT and BDI score is 0.3156. Although technically a positive correlation, the relationship between variables is weak (the nearer the value is to zero, the weaker the relationship). Any value greater than zero are consider a positive correlation, meaning as Y increases, X increases as well. This means when there is increase in OHI-EDENT score which eventually increase MNA and BDI score. Results of the pearson correlation indicated that there is a significant medium positive relationship between Y (OHIEDENT) and X (MNA score), (r (248) = 0.4278, p < .001). The same result is observed between Y (OHIEDENT) and X (BDI score), (r (248) = 0.3156, p < .001) (Figures 1 and 2 - see PDF). This overall finding suggest that the edentulousness has mild to moderate ill effect on nutrition status and psychological status of edentulous patient. These finding were comparatively less in patients using dentures. This may be due to the fact that patients developed a pattern of functioning and adapting their diet to dentures over a period and probably good denture fit and stability of complete denture might have also played a role in improving the chewing ability [5, 15]. However, the success of the treatment is not exclusively related to the technical quality of the prosthesis. The individual's ability to adapt to the new oral condition is an important factor for treatment acceptance, as each patient has different experiences and expectations [16]. One more reason for this suggested by Cousson *et al.* that both groups had insufficient energy intakes and deficits in vitamins and micronutrients; moreover, edentulous subjects had lower intakes than dentate subjects [7]. The MNA and becks inventory finding stated that there no statistical significant of difference between men-female data. Oral health self-perception is a method that assesses the subjective experience of the individual on his oral health, functional, psychological and social well-being. Through the evaluation of oral health-related quality of life (OHRQoL) it is possible to quantify the individuals' perception of the impact of prosthetic treatments on their quality of life [16]. A combination of poor oral health status and unfavourable results for chewing-related OHRQoL items should alert dental professionals to the possibility of nutritional, psychological problems, especially in a population as vulnerable as the elderly. Dietary analysis and psychological counselling should be strictly incorporated into geriatric treatment planning during prosthetic rehabilitation.

## Conclusion:

We show that the self-reported quality of life can be influenced by the tooth loss. There is a chance that edentulousness can negatively affect nutritional and psychological state of individual. Nevertheless, after treatment, the patients reported improved satisfaction with their health states.

## Ethics approval and consent to participate:

An ethical clearance (Under Helsinki declaration) was obtained from ethical Committee COLLEGE OF DENTAL SCIENCE & HOSPITAL, AMARGADH. Written informed Consent was obtained from the each participate.

## Consent for publication:

Not applicable

## Availability of data and materials:

Yes

## Competing interests:

NO

## Funding:

NO

## Authors' contributions:

All the authors have contributed in research and manuscript design.
